# Predicting risk factors for pediatric mortality in clinical trial research: A retrospective, cross-sectional study using a Healthcare Cost and Utilization Project database

**DOI:** 10.1017/cts.2023.634

**Published:** 2023-09-22

**Authors:** Jiahui Ma, Elizabeth A. Johnson, Bernadette McCrory

**Affiliations:** 1 Montana State University, Norm Asbjornson College of Engineering, Bozeman, MT, USA; 2 Montana State University, Mark & Robyn Jones College of Nursing, Bozeman, MT, USA

**Keywords:** Pediatrics, healthcare utilization, clinical trial, big data, safety

## Abstract

**Introduction:**

Incorporating real-world data using “big data” analysis in healthcare are useful to extract specific information for healthcare delivery system improvement. All-cause mortality is an essential measure to enhance patient safety in clinical trial research, especially for underrepresented pediatric participants.

**Objective:**

This study aimed to determine the associations between pediatric mortality and patient-specific factors using the Healthcare Cost and Utilization Project (HCUP) database.

**Methods:**

Data from the 2019 the HCUP Kids’ Inpatient Database (KID) were used to conduct a logistic regression analysis to determine associations between pediatric patients’ the chance of survival and their demographic and socioeconomic background, discharge records, and hospital information.

**Results:**

Total number of diagnoses (OR = 0.84), total number of procedures (OR = 0.86), length of stay (OR = 1.04), age intervals greater than 1 year (OR > 1.0), transfer into the hospital from a different acute care (OR = 0.34), major diagnoses of multiple significant trauma (OR = 0.03) or hepatobiliary system and pancreas (OR = 0.10), region of hospital – west and midwest (OR > 1.0), and medium or larger hospital bed size (OR > 1.0) were all significantly associated with the chance of survival for patients participating in pediatric clinical trials (*p* < 0.05).

**Conclusion:**

Real-world clinical trial data analysis showed the potential improvement area including reallocating trial resources to promote trial quality and safe participation for pediatric patients. Pediatric trials need tools that are developed using user-centered design approaches to satisfy the unique needs and requirements of pediatric patients and their caregivers. Safe intrahospital transfer procedures and active dissemination of successful trial best practices are crucial to trial management, adherence, quality, and safety.

## Introduction

In 2017, the USA had the most clinical trial research participation globally (31% of all clinical trial research participation), which was nearly six times more than participation from the second most participated country [1]. Understanding the US trends in clinical trial research participation can provide enormous insights into global clinical trial research improvement and promote worldwide approval of novel medical interventions (i.e., drugs, devices, techniques, systems, or programs) [1,2].

Participants must consider a multitude of factors prior to enrolling in a clinical trial, such as travel related burden to visit a research location, frequency of scheduled visits, risk inherent with experimental drugs/devices, any direct or indirect benefits, as well as potential adverse events [2]. Compared with adult participants, pediatric clinical trials pose additional ethical and logistical concerns, including conflicts between parents’ concerns with social benefits and obligations, and insufficient knowledge on trial research processes and interventions [2–6]. An additional, but critically important, limiting factor is the availability of a trial due to a perceived or actual lack of return on investment for pharmaceutical companies, particularly for less prevalent or rare conditions/diseases [3,4]. Moreover, the differences in metabolic profiles and weight-dependent dosing beget additional risk for adverse events. Pharmaceutical companies and other trial sponsor groups are less likely to fund or develop drug development programs in the pediatric space [3,4].

Children and adolescents pose different physical, emotional, and social capabilities compared to adults, which requires informed consent and rigorous evaluation of the participant’s ability to complete study procedures involving potential discomfort [3,4]. The experimental product’s effects on pediatric organ development, and volume and frequency of biological specimen collection are common areas of regulatory concern when developing the informed consent and overall protocol. This is often due to a lack of adequate sample size and reliance upon adult studies to inform indications and contraindications in child participants [3–5]. The formal acknowledgment of “therapeutic orphans” for children in clinical trial research calls the attention of improving access for pediatric clinical trial participants across the world [3,4]. Even though some progress has been made [7,8], timely access to pediatric clinical trials is essential for the future development of novel pediatric interventions [3,4]. Recent legislation such as the Research to Accelerate Cures and Equity (RACE) for Children Act of 2017 requires pediatric clinical trials for novel cancer drugs and renews hope and promise to expand pediatric trial access [9]. In response, research sites, academic medical centers, and regulatory boards have called for more literature and evidence in the quantification of pediatric trial participation with a focus on identifying factors which influence safe participation among this vulnerable population [10].

### “Big Data” Approach

With the era of “big data” in healthcare, analyzing and modeling large datasets with advanced statistical tools to better understand associations between patient outcomes and healthcare delivery has demonstrated how to improve healthcare services [11,12]. Incorporating reliable data sources in designing and adjusting clinical trials demonstrated a great opportunity for further improvement, trial generalization, and success [13,14].

### Healthcare Cost and Utilization Project Database

The Healthcare Cost and Utilization Project (HCUP) contains the largest collection of longitudinal hospital care data in the USA, which contains patients’ demographic information, discharge records (diagnosis and procedures received, mortality, severity, and risk evaluation), and enrolled hospital information [15]. Many studies have incorporated the HCUP database to enhance healthcare delivery in clinical trial research, but limited research has studied pediatric clinical trials using the HCUP database [16,17]. The International Classification of Diseases 10^th^ Revision (ICD-10) coding, Z00.6, documents services relate to clinical trial participant examination [18,19], which is in alignment with purpose of the Clinical Treatment Act to expand clinical trial opportunities and benefits to patients and recognize provider effort in delivering clinical trial care activities [20]. All-cause mortality, as an essential indicator for clinical trial research risk, has been widely studied and served as the primary outcome measure from HCUP database in this study [21–23].

### Purpose

The purpose of this study was to determine the associations between mortality and patient-specific factors from the HCUP database. It was hypothesized that patients’ demographic information, discharge records, and enrolled hospital information were associated with the chance of survival in pediatric clinical trials. Understanding and mitigating potential risk factors resulting in pediatric clinical trial mortality would enhance the understanding of safety in the conduct of pediatric clinical trials while further quantifying pediatric participation across multiple disease states through their encounters with hospital systems in the USA.

## Materials and Methods

### Database

The HCUP Kids’ Inpatient Database (KID) includes hospital inpatient pediatric discharge billing data from 1997 to 2019 [24]. The KID contains four discharge-level files, including Core, Severity, Hospital, and Diagnosis and Procedure Groups files [24]. First, the Core file contains patient demographics, expected primary payer, total charges, discharge status, financial status, and the ICD-10 coding for diagnoses and procedures [19]. Second, the Severity file contains additional information on illness severity and mortality risk for each patient’s discharge record [24]. Specifically, measures risk using All Patient Refined Diagnosis Related Group (APRDRG) assigned using software developed by 3 M Health Information Systems [25]. Third, the Hospital file stores characteristics for each hospital participating in the HCUP KID [24]. Finally, the Diagnosis and Procedure Groups file contains additional information on the ICD-10-Clinical Modification (ICD-10-CM) and ICD-10-Procedure Coding System (ICD-10-PCS) [26]. Detailed information on the ICD-10-CM diagnoses and ICD-10-PCS procedures from the Diagnosis and Procedure Groups file was excluded from this study for further research.

In this study, major diagnoses and the clinical trial designation code (i.e., ICD-10 Z00.6 – encounter for examination for normal comparison and control in clinical research program) were considered in the analysis. Analyses were completed using only the 2019 KID data (2019 KID data was the most recent dataset at time of study) to focus specifically on the association between mortality and other factors from KID data [24]. All variables from the Core, Severity, and Hospital files were included in this study.

### Data Sample and Attributes Selection

**Figure 1.**
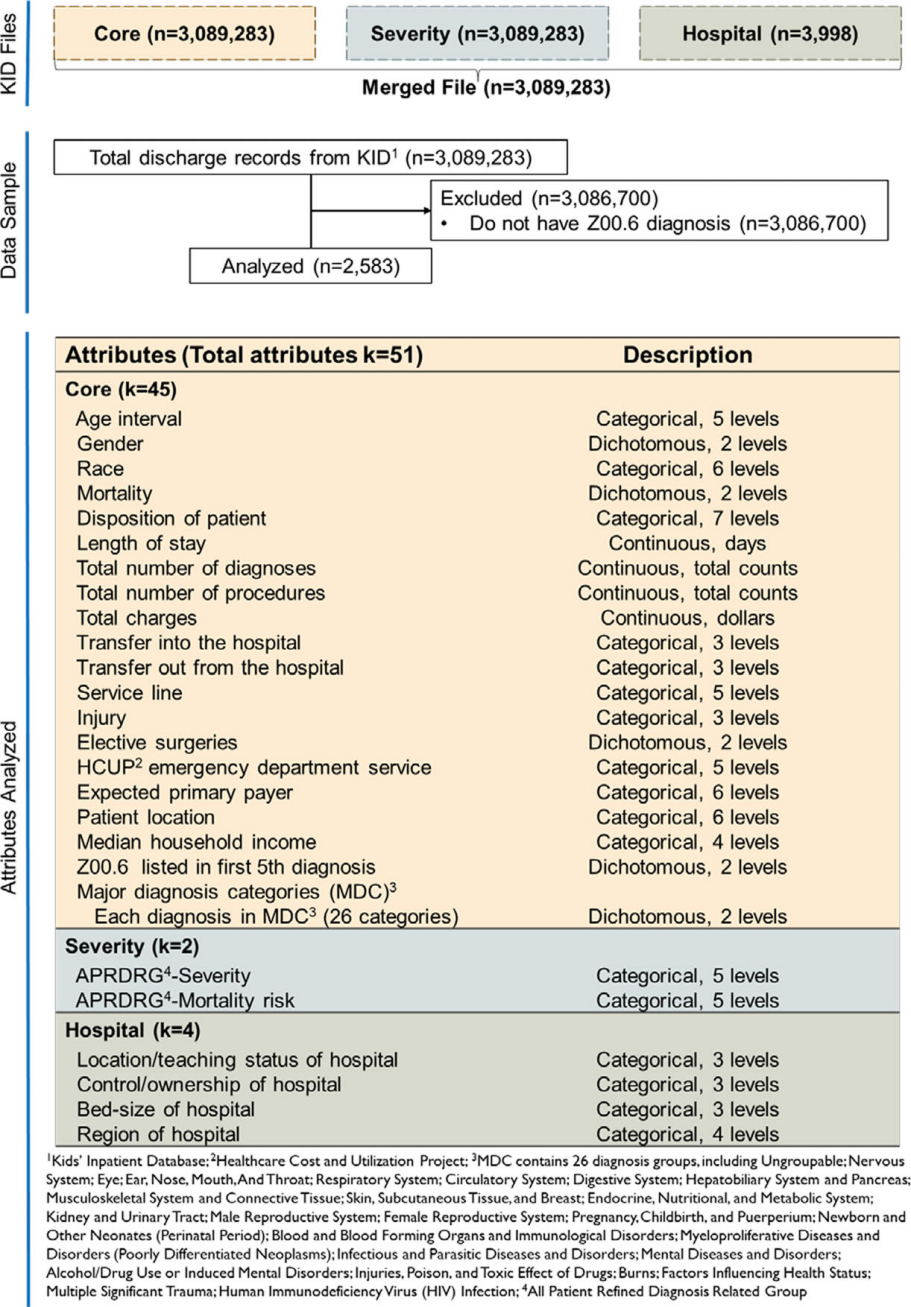
Dataset variables of interest preprocessing.

The Core data file contains 3,089,283 kid discharge records by Record Number (RECNUM) with 129 variables describing demographic information, administrative and discharge, ICD-10-CM diagnoses and procedures information, major diagnosis, insurance, and financial information. Each pediatric inpatient discharge record was connected to the Hospital data by HCUP KID hospital number (HOSP_KID). The Hospital file contains information on the region, teaching status, ownership, and bed size of the 3,998 participating hospitals [24]. The Severity file has illness severity and mortality risk information for 3,089,283 kid discharge records identified by RECNUM and HOSP_KID that were linked to the Core and Hospital files. The Severity and Hospital file were merged with the Core file by identifier RECNUM and HOSP_KID for each discharge record (Fig. 1).

The ICD-10 code is classified and assigned to each discharge record on their diagnoses/qualifying care activities and procedures. To understand the clinical trial-related activities, discharge records with the Z00.6 ICD-10 code, associated with clinical trial visits or procedures for control or interventional participants, were extracted from the merged dataset that included the Core, Severity, and Hospital datasets for analyses of the 3,089,283 records only 2,583 included Z00.6 as a diagnosis and therefore 3,086,700 records were excluded from this current analysis (Fig. 1).

Fifty-one attributes were analyzed in this study (Fig. 1). Admission information (i.e., day, month, newborn birth, and neonatal age), detailed ICD-10 diagnoses (i.e., other ICD-10 diagnoses except for Z00.6), ICD-10 procedure information (i.e., count of procedures and number of days, procedure applied), and hospital information (i.e., location, ownership, region, and bed size) were excluded from the analysis. A new variable was created to determine if Z00.6 was listed in the first five diagnoses among all diagnoses in the inpatient record. The final selected variables for the analysis were summarized in Fig. 1. Major diagnosis categories (MDC) were regrouped into 26 mutual exclusive categories from all diagnoses from the ICD-10 diagnoses. The number of diagnoses for each category varied largely from each other. To balance the dataset and reduce the numbers of levels in MDC attribute for statistical modeling, MDC was converted into 26 individual variables with each variable containing two levels (1 indicating that participants were diagnosed with the corresponding MDC category, and 0 indicating that participants were not diagnosed with the corresponding MDC category).

Due to skewness, age was converted into an interval variable with five intervals ranging from 0, 1–4, 5–9, 10–14, and 15–20 (Table 1). To compare the differences among all participants in a clinical trial *(n* = 2,583) and deceased participants also in a clinical trial (*n* = 35), the demographic, insurance, financial, and hospital information were summarized (Table 1). Deceased participants tended to be younger, included more females, and represented a larger proportion of underrepresented minority groups compared with all clinical trial participants. There were no differences with respect to insurance, primary payer, and location among either clinical trial participant group. While clinical trial participants were equally likely to come from all income levels, a higher proportion of deceased participants were from the lower income households ($47,999 or less per year) (42.9%). Urban teaching hospitals (99.3%) and nonprofit private organizations (74.4%) hospitals participated most frequently with clinical trial participants. Hospitals from the southern region (including 17 states) included the largest frequency of participants with the Z.006 diagnosis (33.4%). However, there were more deceased participants from the northeast region (28.6%), which had the lowest Z.006 diagnosis proportion of all regions. Most participants received care in large hospital facilities [27]. However, deceased participants most frequently had an inpatient stay at a small hospital facilities when morbidity occurred [27].

**Table 1.** Clinical trials participants and hospital information summary

Variable	Overall Z00.6 (*n* = 2583)	Mortality (*n* = 35)
**Demographic information**		
Age interval, years*		
0 (less than 12 months)	645 (25.0%)	16 (45.7%)
1–4	589 (22.8%)	7 (20.0%)
5–9	326 (12.6%)	1 (2.9%)
10–14	404 (15.6%)	5 (14.3%)
15–20	619 (24.0%)	6 (17.1%)
Female*	1181 (45.7%)	18 (51.4%)
Race/ethnicity*		
White	1360 (52.7%)	9 (25.7%)
Black	349 (13.5%)	11 (31.4%)
Hispanic	385 (14.9%)	6 (17.1%)
Asian or Pacific Islander	95 (3.7%)	1 (2.8%)
Native American	9 (0.3%)	–
Other	151 (5.8%)	2 (5.7%)
**Insurance and financial information**		
Expected primary payer*		
Medicare	5 (0.2%)	–
Medicaid	1133 (43.9%)	19 (54.3%)
Private insurance	1195 (46.3%)	10 (28.6%)
Self-pay	51 (2.0%)	3 (8.6%)
No charge	–	–
Other	178 (6.9%)	3 (8.6%)
Patient location (NCHS Urban-Rural Code)* ^,^ **		
“Central” counties of metro areas of >= 1 million pop.	785 (30.4%)	15 (42.9%)
“Fringe” counties of metro areas of >= 1 million pop.	651 (25.2%)	9 (25.7%)
Counties in metro areas of 250,000–999,999 pop.	540 (20.9%)	8 (22.9%)
Counties in metro areas of 50,000–249,999 pop.	236 (9.2%)	3 (8.6%)
Micropolitan counties	203 (7.9%)	–
Not metropolitan or micropolitan counties	162 (6.3%)	–
Median household income (for patient’s ZIP code)*		
0–25th percentile	706 (27.3%)	15 (42.9%)
26th–50th percentile	602 (23.3%)	8 (22.9%)
51th–75th percentile	620 (24.0%)	10 (28.6%)
76th–100th percentile	631 (24.4%)	2 (5.7%)
**Hospital information**		
Location/teaching status of hospital*		
Rural	–	–
Urban, nonteaching	17 (0.7%)	1 (2.9%)
Urban, teaching	2566 (99.3%)	34 (97.1%)
Control/ownership of hospital*		
Government, non-federal	553 (21.4%)	4 (11.4%)
Private, not-for-profit	1921 (74.4%)	30 (85.7%)
Private, investor-owned	109 (4.2%)	1 (2.9%)
Region of hospital*		
Northeast	515 (19.9%)	10 (28.6%)
Midwest	540 (20.9%)	9 (25.7%)
South	862 (33.4%)	11 (31.4%)
West	666 (25.8%)	5 (14.3%)
Bed size of hospital*		
Small	199 (7.7%)	6 (17.1%)
Medium	320 (12.4%)	1 (2.9%)
Large	2064 (79.9%)	28 (80%)

* Frequency (relative frequency).

** National Center for Health Statistics.

### Data Analysis and Modeling

Both descriptive and inferential analyses were completed using R programming language (version R-4.3.0) [28] and Tidyverse and ggplot2 packages [29,30]. Mean, standard deviation, and median were calculated for all continuous variables, and counts and relative frequency were calculated for all categorical variables. ANOVA and Chi-square analysis were applied as inferential analysis to find variable associations.

Filter and wrapper methods were two major feature selection methods to reduce the computation time and improve model prediction performance for statistical analysis and machine learning models [31]. Pearson’s correlations [32] and Cramer’s V values [33] were the filter feature selection method, and both were calculated for the association strength among variables to select predictors for the logistic regression model [34].

The logistic model used survival (yes and no) as the dependent variable while including each selected feature after the filter feature selection method applied to predictors (see Fig. 1) for pediatric clinical trials involvement as independent variables. Selected predictors were individually fitted in the univariate logistic regression model. Significant predictors (with p-value less than significance level) were selected to fit a multivariate logistic regression with mortality as response variable. The backward elimination selection method as one popular wrapper feature selection method was used to select the best predictors for the logistic regression model [31]. The backward elimination removed the predictors with the largest insignificant p-value recursively until all predictors in the multivariate logistic regression model were with a p-value less than the significance level [35]. The significance level for all statistical analyses in this study was 0.05.

## Results

### Prevalence of Participants with Clinical Trial Diagnosis (Z00.6)

All clinical trial participants’ and deceased participants’ inpatient characteristics were summarized in Table 2. The proportion of the overall population who had the clinical trial Z00.6 code listed within their first five diagnoses (50.9%) was two times the proportion of deceased participants (25.7%). Deceased participants tended to have a greater count or number of total diagnoses (*p* < 0.001), received more procedures (*p* < 0.001), and stayed longer in the hospital (*p* = 0.07) than the overall clinical trial participants. Deceased participants (11.4%) received a significantly less proportion of elective surgeries compared to the overall clinical trial participants (38.2%) (*p* = 0.003). Most trial participants reported no injuries (96.6% for all participants and 97.1% for deceased participants), which included displaced transverse fracture and burning. One mortality case was due to an injury. Most participants (83%) visited the hospital for routine treatment. Over half of all clinical trial participants used a medical service line (54.6%) during hospital visits, but deceased participants used more maternal and neonatal (34.3%) and surgical (34.3%) service lines than medical service lines (28.6%). Most of the deceased participants did not use the emergency room (91.4%). Nearly half of the deceased participants (45.7%) were transferred into the hospital, most often from an acute care hospital (42.9%). Deceased participants experienced an extremely high frequency of function loss (71.4%) versus the overall clinical trial participants (16.1%).

**Table 2.** Inpatient characteristics summary

Variable	Overall Z00.6 (*n* = 2583)	Mortality (*n* = 35)
Z00.6 listed in first 5th diagnosis*	1315 (50.9%)	9 (25.7%)
Length of stay (days)**	19.9 (5, 34.1)	30.3 (14, 46.2)
Total number of diagnoses (count)**	11.7 (10, 7.2)	20.5 (21, 7.0)
Total number of procedures (count)**	3.9 (2, 4.7)	9.9 (8, 6.6)
Total charges (US dollars)**	$274,558 (76,619, 598,228)	$765,445 (336,331, 978,308)
Elective surgery (count)*	986 (38.2%)	4 (11.4%)
Injury (incidence)*		
No injury	2496 (96.6%)	34 (97.1%)
Injury is reported in first-listed diagnosis	37 (1.4%)	1 (2.9%)
Injury is reported other than the first-listed diagnosis	50 (2.0%)	–
Disposition of patient*		
Routine	2155 (83.4%)	–
Transfer to short-term hospital	57 (2.2%)	–
Transfer other	60 (2.3%)	–
Home health care	269 (10.4%)	–
Against medical advice	2 (0.1%)	–
Died in hospital	35 (1.4%)	35 (100%)
Discharge alive (destination unknown)	3 (0.1%)	–
Service line (based on ICD-10)* ^,^ ***		
Maternal and neonatal	576 (22.3%)	12 (34.3%)
Mental health/substance use	15 (0.6%)	–
Injury	37 (1.4%)	1 (2.8%)
Surgical	544 (21.1%)	12 (34.3%)
Medical	1411 (54.6%)	10 (28.6%)
Transfer into the hospital*		
Not transferred in or newborn admission	2131 (82.5%)	19 (54.3%)
Transferred in from a different acute care hospital	414 (16%)	15 (42.9%)
Transferred in from another type of health faculty	28 (1.1%)	1 (2.9%)
Transfer out from the hospital*		
Not a transfer	2464 (95.4%)	35 (100%)
Transferred out to a different acute care hospital	57 (2.2%)	–
Transferred out to another type of health faculty	60 (2.3%)	–
Risk mortality*		
No class specified	5 (0.2%)	–
Minor likelihood of dying	1050 (40.7%)	3 (8.6%)
Moderate likelihood of dying	989 (38.3%)	3 (8.6%)
Major likelihood of dying	380 (14.7%)	6 (17.1%)
Extreme likelihood of dying	159 (6.2%)	23 (65.7%)
Risk severity*		
No class specified	5 (0.2%)	–
Minor loss of function	414 (16.0%)	3 (8.6%)
Moderate loss of function	990 (38.3%)	2 (5.7%)
Major loss of function	758 (29.3%)	5 (14.3%)
Extreme loss of function	416 (16.1%)	25 (71.4%)
ED record*		
Record does not meet HCUP ED criteria	2021 (78.2%)	32 (91.4%)
ED revenue code on record	432 (16.7%)	3 (8.6%)
Positive ED charge	127 (4.9%)	–
ED CPT procedure code on record	–	–
Condition code P7 indication of ED admission, point of origin of ED, or admission source of ED	3 (0.1%)	–

CPT = current procedural terminology; ED = emergency department; HCUP = healthcare cost and utilization project.

* Mean (median, standard deviation).

** Frequency (relative frequency).

*** International Classification of Diseases 10th Revision.

**Figure 2.**
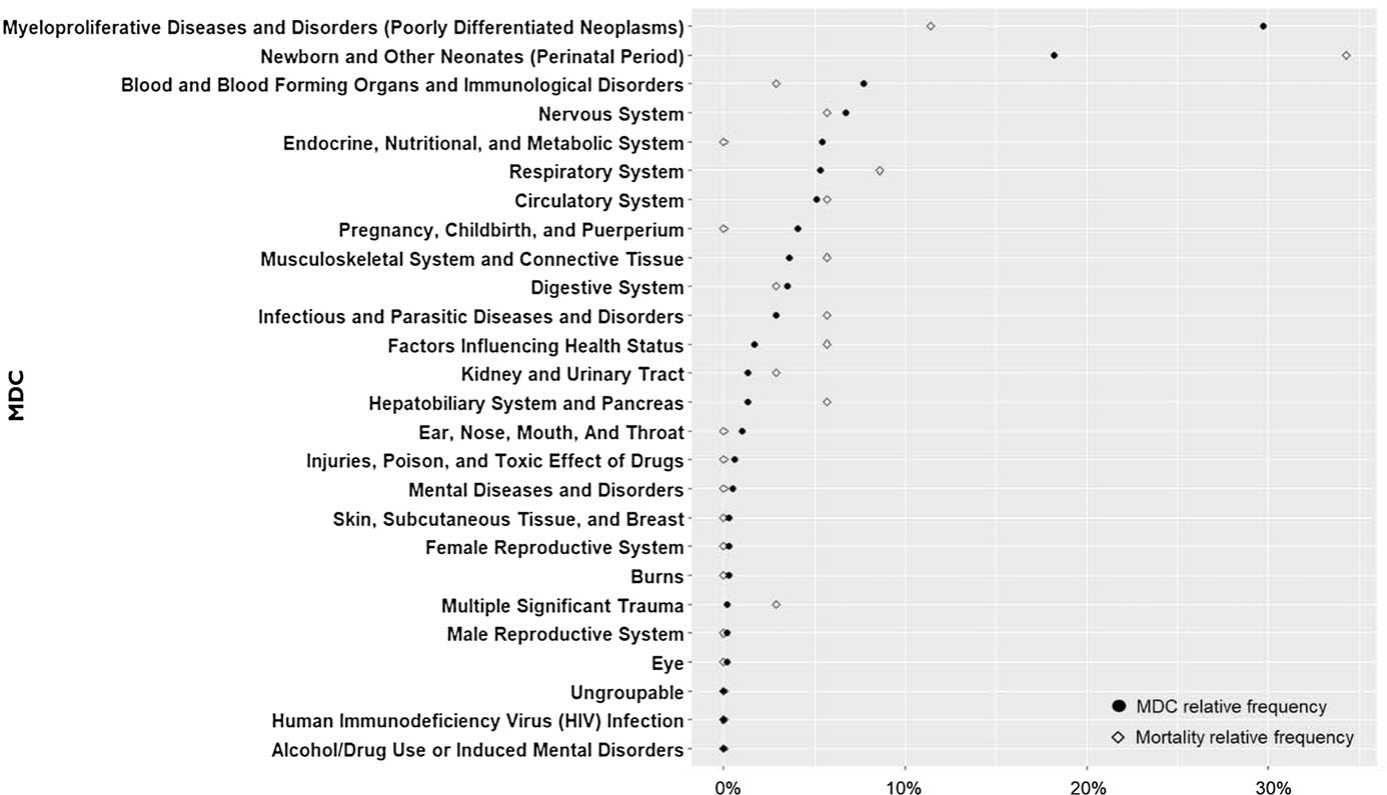
Major diagnosis categories (MDC) relative frequency compared with mortality relative frequency.

MDC relative frequency is calculated by the number of each MDC category’s frequency over the total number of participants (*n* = 2583) in clinical trials, and mortality relative frequency is calculated by the number of deceased participants for each MDC category over the total number of deceased participants (*n* = 35) (Fig. 2). Myeloproliferative Diseases and Disorders, and Newborn and Other Neonates were the most frequent major diagnoses compared with other diagnoses and had a mortality rate greater than 10%. Myeloproliferative Diseases and Disorders had a lower mortality rate than its MDC relative frequency, but Newborn and Other Neonates had a higher mortality rate than its MDC relative frequency. Other major diagnoses were less frequently diagnosed and mortality in participants (<10%).

### Factors Associated with Participants Mortality

#### Filter feature selection for logistic regression

Pearson’s correlation analysis reviewed significant correlations (*p*-value < 0.05) between continuous variables. Length of stay, the total number of diagnoses, and the total number of procedures were moderately correlated with each other (*r* < 0.7). Total charges and length of stay had a correlation of 0.7 which can cause potential collinearity in the logistic regression. Total charges had a larger scale than the other three continuous variables. Thus, length of stay, the total number of diagnoses, and the total number of procedures were included in the logistic regression.

Categorical variables summarized in Fig. 1 were considered in the logistic regression. Cramer’s V value was calculated for each pair of categorical variables to observe the association between them. Most of the associations were below 0.5 except for the association between service line with injury (0.71) and Newborn and Other Neonates (0.88), and between age interval and Newborn and Other Neonates diagnosis (0.82). Therefore, service line and Newborn and Other Neonates diagnosis were excluded from the logistic regression model.

#### Logistic regression model

The final multivariate logistic regression model was summarized in Table 3. The decrease in the total number of diagnoses and the total number of procedures caused higher odds of survival (OR<1, *p* < 0.001), and the longer length of stay tended to raise the odds of survival (OR>1, *p* < 0.001). Participants transferred in from a different acute care hospital had lower odds (OR = 0.34 < 1) of survival than not transferred participants (p = 0.001). Participants registered in the hospital from midwest (OR = 6.86, *p* = 0.004) and west (OR = 4.63, *p* = 0.015) had higher odds of survival than participants from the northwest hospital. Participants aged from 1 to 4 years (OR = 5.29, *p* = 0.009) and above 15 years (OR = 2.42, *p* = 0.002) tended to have a higher chance of survival than newborns (age<1). Participants used the larger bed size (median and large) had higher chance of survival than participants used the small bed size (OR<1, *P* < 0.05). Participants diagnosed with multiple significant traumas (OR = 0.03, *p* = 0.019) and hepatobiliary system and pancreas disorders (OR = 0.10, *p* = 0.010) tended to have a lower chance of survival compared to other participants.

**Table 3.** Logistic regression results summary for participants mortality

Factor^a^	Odds ratios for predictors			Coefficients^b^	
Level A	Level B^c^	OR	95% CI	*β*	*P*-value
Total number of diagnoses^d^	–	0.84	(0.77, 0.90)	−0.18	<0.001**
Total number of procedures^d^	–	0.86	(0.79, 0.93)	−0.15	<0.001**
Length of stay^d^	–	1.04	(1.02, 1.06)	0.04	<0.001**
Age interval, years					
0 (less than 12 months)	1–4	5.29	(1.61, 20.66)	1.66	0.009**
	5–9	8.61	(1.47, 167.89)	2.15	0.05
	10–14	1.77	(0.56, 6.30)	0.57	0.345
	15–20	11.2	(2.76, 60.63)	2.42	0.002**
Transfer into the hospital					
Not transferred in or newborn admission	Transferred in from a different acute care hospital	0.34	(0.14, 0.88)	−1.07	0.021*
	Transferred in from another type of health faculty	0.58	(0.09, 11.64)	−0.54	0.631
Multiple significant trauma					
Did not diagnose	Diagnosed	0.03	(0.002, 0.95)	−3.45	0.019*
Hepatobiliary system and pancreas					
Did not diagnose	Diagnosed	0.1	(0.02, 0.76)	−2.34	0.010*
Region of hospital					
Northeast	Midwest	6.86	(1.95, 26.61)	1.93	0.004**
	South	2.08	(0.67, 6.54)	0.73	0.202
	West	4.63	(1.39, 17.24)	1.53	0.015*
Bed size of hospital					
Small	Medium	22.84	(2.82, 513.34)	3.13	0.012*
	Large	3.75	(1.16, 10.94)	1.32	0.019*

a Only statistically significant factors/levels and near/close to be significant factors and their levels were listed in the table.

b Coefficients of level A: *P-value < 0.05; **P-value < 0.01.

c Odd ratios for level A to level B.

d The median of the number of diagnoses is 10, the median of the number of procedures is 2, and the median of length of stay is 5.

## Discussion

All-cause mortality has been widely used to measure healthcare delivery success as an end point, but the cause of all-cause mortality is usually difficult and complicated to determine with restricted data resources [36,37]. However, factors associated with the mortality or chance of survival can shed light on the potential causes for death and show areas for caution/improvement in pediatric clinical trials. This study contributed by modeling nationwide KID data [24] with ICD-10 code Z00.6 (encounter for examination for normal comparison and control in clinical research program) to understand the potential risks of mortality and increase the success of pediatric clinical trial research.

### Demographic Information

Nine factors including patients’ demographic information, discharge records, and enrolled hospital information had a significant effect on patients’ chance of survival (Table 3). It is remarkable that a quarter of the children who participated in clinical trial research were less than 12 months old (Table 1). Compared to infants, children older than 12 months had a higher chance of survival. Babies, children aged from 1 to 4 years, and teenagers aged 15–18 years had significantly greater chance of survival (*p* < 0.05, Table 3). Infants were less likely to survive in trials, which may be due to their premature organ development and subdued immunity system [38]. The medical complexity of infants demonstrates the crucial need for heightened provider awareness of research activities [39], particularly when affecting clinical decision-making (e.g., medication dosing and nursing assessments) [40]. Children, especially infants, may find discomfort or difficulty interacting with the technology and devices designed for the adult population often required in clinical research. User-centered design could be incorporated in clinical trial research to address the physical and cognitive limitations often posed in this vulnerable population [41]. Additionally, user-centered developed approaches and tools would assist caregivers and healthcare professionals in trial adherence, communication, decision-making, and engaged participation [42]. Participatory design involving all key stakeholders of tools, like web-based applications, has the potential to dramatically enhance trial quality and mitigate risk [43].

### Discharge Record

Patients with a greater number of diagnoses (OR = 0.84) and procedures (OR = 0.86) tended to have more symptoms and complicated treatments [44], which was associated with a lower chance of survival (*p* < 0.001). Even though length of stay was moderately correlated with number of diagnoses and procedures (0.5 < *r* < 0.7, *p* < 0.05), the longer time of staying in hospital was associated with a higher chance of survival (OR = 1.04, *p* < 0.001). This association was also contrasted with findings from adult patients [36,45], which showed the magnitude separating children from adult clinical trial research.

Patients transferred in from a different acute hospital were less likely to survive compared to patients not transferred (*p* < 0.05, Table 3). Patients transferred between hospitals were more critically ill and needed more advanced treatment [46]. Intrahospital transfer elements, such as decision to transfer and communication, pretransfer stabilization and preparation, ways to transfer, and documentation for receiving facilities could be better designed to enhance survival outcomes [46–48].

Myeloproliferative Diseases and Disorders had the highest mortality rate compared with other diagnoses. Yet, multiple significant traumas and hepatobiliary system and pancreas disorders were the only two diagnoses significantly associated with a decreased chance of survival for pediatric clinical trial participants (*p* < 0.05, Table 3). This is not surprising since there has been growing participation in oncology trials and higher survival rate for children with cancer in recent years [3,4]. Children diagnosed with cancer have more access to better managed clinical trials compared with children diagnosed with other diseases. Protocols in oncology trials may be a useful reference for other disease trials to improve the overall survival rate in pediatric clinical trials.

### Enrolled Hospital Information

Hospitals from the west and midwest had a significantly higher chance of survival for pediatric clinical trial participants compared with hospitals from the northeast (*p* < 0.05, Table 3). The study showed that a lack of healthcare professionals, such as physicians and registered nurses, was associated with a higher mortality rate in acute care hospitals [49]. The shortage of healthcare professionals may be a major reason to explain a lower chance of survival for pediatric clinical trial research in the northeast and a higher chance of survival in the west and midwest. The number of short-term acute care beds (hospital bed size) was positively associated with the chance of survival for pediatric clinical trial patients (*p* = 0.012 for medium bed size & *p* = 0.019 for large bed size, Table 3). Hospitals with medium and large bed sizes tended to have a higher chance of survival compared to those with a small bed size. Unlike hospitals in other high-income countries [50], inpatient care in hospitals with smaller bed sizes contributed to a lower death risk for pediatric clinical trial patients [50].

In this study, multifaceted factors including patient health conditions, diagnoses, treatment procedures, socioeconomic status, as well as hospital locations and resources were modeled for the all-cause mortality among pediatric clinical trial patients. Incorporating several datasets into a larger dataset (i.e., big data) enabled advanced statistical analyses and uncovered potential root causes to all-cause pediatric mortality. Clinical trials often represent an alternative to standard of care treatment for many patients, particularly for those seeking options after existing therapies have been exhausted or proved ineffective. These patients often require frequent visits and procedures over long periods of time in disparate research locations away from familial support structures. Through the use of big data, patterns of research integration process gaps may be uncovered to support this unique population of research participants which straddle both investigational and clinical realms of healthcare delivery.

### Limitations

The KID database includes only hospital discharge data, which is not exclusive to patient care in a clinical trial, making interpretation of the clinical trial patient experience difficult. Promising factors associated with the chance of survival for pediatric clinical trials were determined; however, additional variables (e.g., detailed ICD-10-CM diagnoses and procedures in KID dataset) were excluded from this study. Also, the KID data used in this study was only from a single year’s data (2019). Future studies are needed to include added diagnoses and procedures variables from the most recent data available. Lastly, a prospective longitudinal study following a cohort of rural pediatric clinical trial participants would allow linking outcomes, safety, and patient-reported outcome measures to holistically develop tailored best practices for pediatric clinical trial management.

## Conclusion

This study provides insights into understanding and mitigating potential risk factors resulting in pediatric clinical trial all-cause mortality using the 2019 HCUP KID dataset. Results from this study draw attention to safety concerns of pediatric trial patients in hospital settings. Total number of diagnoses, total number of procedures, length of stay, age, hospital transfer, major diagnosis of multiple significant trauma or hepatobiliary system and pancreas, hospital region, and bed size were all significantly associated with chance of survival for patients who participated in pediatric clinical trials. These nine significant factors impacting chance of survival uncovered areas which need additional study and validation for clinical trial sponsors and associated healthcare facilities to create best practices for pediatric clinical trial management. This study’s unique focus on exclusively pediatric patients highlighted the risk of this vulnerable population’s morbidity and mortality in clinical trials. Expansion of trial participation via legislation means renewed focus and attention to trial participation across rural settings, as well as a need to ensure research professional capacity/training among all types of clinicians. With the popularity of decentralized and hybrid clinical trial models, there is a higher likelihood of a clinical provider encountering a trial participant for emergent or urgent care needs and as part of interfacility transfers. Safe intrahospital transfer protocols and efficacious information transfer tools are essential to ensure patient safety and quality. Learning from successful clinical trial design, such as oncology trials, may be a starting strategy to enhance protocol design and legislation for other clinical trial research including pediatrics. Awareness of the unique care required with clinical trial participants can not only be achieved through clinical professional training but also leveraging electronic health record platform functionalities to guide and automate research safety information availability during clinical decision-making. Assimilating real-world clinical trial data from multiple sources and using the latest in “big data” analysis techniques could lead to better and nearly real-time oversight and monitoring of adherence, effectiveness, and outcomes, especially for underrepresented groups. Future research will apply machine learning and data mining models to find additional associations for all-cause mortality in pediatric clinical trial research using additional data years to evaluate changes and trends.

## References

[1] U.S. Food & Drug Administration. *Global Participation in Clinical Trials Report 2015-2016*. 2017. https://www.fda.gov/media/106725/download

[2] Pawlik TM , Sosa JA. Clinical trials. In: Success in Academic Surgery. 2nd ed. London: Springer International Publishing, 2020.

[3] Caldwell PHY , Murphy SB , Butow PN , Craig JC. Clinical trials in children. Lancet. 2004;364(9436):803–811. doi: 10.1016/S0140-6736(04)16942-0. 15337409

[4] Joseph PD , Craig JC , Caldwell PHY. Clinical trials in children. Br J Clin Pharmacol. 2015;79(3):357–369. doi: 10.1111/bcp.12305. 24325152 PMC4345947

[5] Shaddy RE , Denne SC. Clinical report--guidelines for the ethical conduct of studies to evaluate drugs in pediatric populations. Pediatrics. 2010;125(4):850–860. doi: 10.1542/peds.2010-0082. 20351010

[6] Gul RB , Ali PA. Clinical trials: the challenge of recruitment and retention of participants. J Clin Nurs. 2010; 19(1-2):227–233. doi: 10.1111/j.1365-2702.2009.03041.x. 20500260

[7] de Rojas T , Pearson AJ , Scobie N , et al. Intercontinental collaboration in clinical trials for children and adolescents with cancer—A systematic review by ACCELERATE. Cancer Med. 2021;10(23):8462–8474. doi: 10.1002/cam4.4356. 34687165 PMC8633236

[8] Steinbrook R. Testing medications in children. N Engl J Med. 2002;31(18):1462–1470. doi: 10.1056/NEJMhpr021646. 12409558

[9] Zettler ME. The RACE for children act at one year: progress in pediatric development of molecularly targeted oncology drugs. Expert Rev Anticancer Ther. 2022;22(3):317–321. doi: 10.1080/14737140.2022.2032664. 35051348

[10] Ittenbach RF , Corsmo JJ , Kissling AD , Strauss AW. How many minors are participating in clinical research today? An estimate and important lessons learned. J Clin Transl Sci. 2021;5(1):e179. doi: 10.1017/cts.2021.844. 34849254 PMC8596071

[11] Ngiam KY , Khor IW. Big data and machine learning algorithms for health-care delivery. The Lancet Oncology. 2019;20(5):e262–e273. doi: 10.1016/S1470-2045(19)30149-4. 31044724

[12] Sridhar S , Whitaker B , Mouat-Hunter A , McCrory B. Predicting length of stay using machine learning for total joint replacements performed at a rural community hospital. PLOS ONE. 2022;17(11):e0277479. doi: 10.1371/journal.pone.0277479. 36355762 PMC9648742

[13] He Z , Tang X , Yang X , et al. Clinical trial generalizability assessment in the big data era: a review. Clin Translat Sci. 2020;13(4):675–684. doi: 10.1111/cts.12764. PMC7359942 32058639

[14] Mayo CS , Matuszak MM , Schipper MJ , Jolly S , Hayman JA , Ten Haken RK. Big data in designing clinical trials: opportunities and challenges. Methods. Front Oncol. 2017; 7:187. doi: 10.3389/fonc.2017.00187. 28913177 PMC5583160

[15] HCUP Kids’ Inpatient Database (KID) 1997–2019. Healthcare Cost and Utilization Project (HCUP). Agency for Healthcare Research and Quality, Rockville, MD. www.hcup-us.ahrq.gov/kidoverview.jsp

[16] Yu JS , Sanchez L , Zeitlin J , Sosa B , Sculco P , Premkumar A. Characterization and potential relevance of randomized controlled trial patient populations in total joint arthroplasty in the United States: a systematic review. J Arthroplasty. 2022;37(12):2473–2479. doi: 10.1016/j.arth.2022.06.010. 35750151

[17] Qureshi AI , Singh B , Huang W , et al. Mechanical thrombectomy in acute ischemic stroke patients performed within and outside clinical trials in the United States. Neurosurgery. 2020;86(1):E2–E8. 10.1093/neuros/nyz359 31670379

[18] Maccariella-Hafey P. Code Z00.6 and Clinical Trials. Health Information Associates. https://hiacode.com/blog/education/clinical-trials. Accessed June 24th, 2023.

[19] Healthcare Cost and Utilization Project (HCUP). HCUP Clinical Classifications Software Refined (CCSR) for ICD-10-CM diagnoses, v2021.2. Rockville, MD: Agency for Healthcare Research and Quality. www.hcup-us.ahrq.gov/toolssoftware/ccsr/dxccsr.jsp. 21413206

[20] Clinical Treatment Act, S.4742, 116th Congress (2019–2020). https://www.congress.gov/bill/116th-congress/senate-bill/4742.

[21] van Eijk RPA , Roes KCB , de Greef-van der Sandt I , van den Berg LH , Lu Y. Functional loss and mortality in randomized clinical trials for amyotrophic lateral sclerosis: to combine, or not to combine—that is the estimand. Clin Pharmacol Ther. 2022;111(4):817–825. doi: 10.1002/cpt.2533. 35076930 PMC8940672

[22] Becerra-Tomás N , Blanco Mejía S , Viguiliouk E , et al. Mediterranean diet, cardiovascular disease and mortality in diabetes: a systematic review and meta-analysis of prospective cohort studies and randomized clinical trials. Crit Rev Food Sci. 2020;60(7):1207–1227. doi: 10.1080/10408398.2019.1565281. 30676058

[23] Mentz RJ , Anstrom KJ , Eisenstein EL , et al. Effect of torsemide vs furosemide after discharge on all-cause mortality in patients hospitalized with heart failure: the TRANSFORM-HF randomized clinical trial. JAMA. 2023;329(3):214–223. doi: 10.1001/jama.2022.23924. 36648467 PMC9857435

[24] HCUP Kids’ Inpatient Database (KID). Healthcare Cost and Utilization Project (HCUP). Rockville, MD: Agency for Healthcare Research and Quality, 2019; www.hcup-us.ahrq.gov/kidoverview.jsp.

[25] Averill RF , Goldfield N , Muldoon J , et al. A closer look at all-patient refined DRGs. J AHIMA. 2002;73(1):46–50. 12469662

[26] Centers for Medicare and Medicaid Services. ICD-10-CM/PCS MS-DRG v37.0 Definitions Manual. n.d. https://www.cms.gov/icd10m/version37-fullcode-cms/fullcode_cms/P0001.html 26110197

[27] HCUP KID Description of Data Elements. The Healthcare Cost and Utilization Project. https://hcup-us.ahrq.gov/db/vars/hosp_bedsize/kidnote.jsp. Accessed June 1, 2023.

[28] R Core Team. R: A language and environment for statistical computing. *R Foundation for Statistical Computing*. Vienna, Austria. 2018; http://www.R-project.org/

[29] Wickham H , Averick M , Bryan J , et al. Welcome to the tidyverse. J Open Source Softw. 2019;11(21):1686. doi: 10.21105/joss.01686.

[30] H W. ggplot2: Elegant Graphics for Data Analysis. Springer-Verlag New York. 2016;

[31] Chandrashekar G , Sahin F. A survey on feature selection methods. Comput Electr Eng. 2014;40(1):16–28. doi: 10.1016/j.compeleceng.2013.11.024.

[32] Mukaka MM. Statistics corner: a guide to appropriate use of correlation coefficient in medical research. Malawi Med J. 2012;24(3):69–71. 23638278 PMC3576830

[33] Allen M. The SAGE Encyclopedia of Communication Research Methods. Thousand Oaks, California: SAGE Publications; 2017.

[34] Cramer JS. The Origins of Logistic Regression, Rotterdam, Netherlands: Tinbergen Institute; 2002.

[35] Borboudakis G , Tsamardinos I. Forward-backward selection with early dropping. J Mach Learn Res. 2019;20(1):276–314.

[36] Friedrich JO , Harhay MO , Angus DC , et al. Mortality as a measure of treatment effect in clinical trials recruiting critically ill patients*. Crit Care Med. 2023;51(2):222–230. 36661450 10.1097/CCM.0000000000005721

[37] Eisenstein ECL , Prather K , et al. Rethinking Clinical Trials: A Living Textbook of Pragmatic Clinical Trials. Choosing and Specifying Endpoints and Outcomes: Using Death as an Endpoint. Bethesda, MD: NIH Pragmatic Trials Collaboratory; 2022.

[38] Kimla K , Nathanson D , Woolfenden S , Zwi K. Identification of vulnerability within a child and family health service. Aust Health Rev. 2019;43(2):171–177. doi: 10.1071/ah17024. 29157354

[39] Comis RL , Miller JD , Colaizzi DD , Kimmel LG. Physician-related factors involved in patient decisions to enroll onto cancer clinical trials. J Oncol Pract. 2009;5(2):50–56. doi: 10.1200/jop.0922001. 20856718 PMC2790653

[40] Kloc M , Ghobrial RM , Kuchar E , Lewicki S , Kubiak JZ. Development of child immunity in the context of COVID-19 pandemic. Clin Immunol. 2020;217:108510. doi: 10.1016/j.clim.2020.108510. 32544611 PMC7293525

[41] Walden A , Garvin L , Smerek M , Johnson C. User-centered design principles in the development of clinical research tools. Clin Trials. 2020;17(6):703–711. doi: 10.1177/1740774520946314. 32815381

[42] Fleisher L , Bass SB , Shwarz M , et al. Using theory and user-centered design in digital health: the development of the mychoice communication tool to prepare patients and improve informed decision making regarding clinical trial participation. Psycho-Oncol. 2020;29(1):114–122. doi: 10.1002/pon.5254. 31654442

[43] Shikako K , Mogo ERI , Grand-Maison V , Simpson R , Pritchard-Wiart L , Majnemer A. Designing user-centered mobile health initiatives to promote healthy behaviors for children with disabilities: development and Usability study. JMIR Form Res. 2021;5(9):e23877. doi: 10.2196/23877. 34528886 PMC8485194

[44] Fried LP , Storer DJ , King DE , Lodder F. Diagnosis of illness presentation in the elderly. J Am Geriatr Soc. 1991;39(2):117–123. doi: 10.1111/j.1532-5415.1991.tb01612.x. 1991942

[45] Fanaroff AC , Clare R , Pieper KS , et al. Frequency, regional variation, and predictors of undetermined cause of death in cardiometabolic clinical trials: a pooled analysis of 9259 Deaths in 9 trials. Circulation. 2019;139(7):863–873. doi: 10.1161/CIRCULATIONAHA.118.037202. 30586739 PMC6370494

[46] Kulshrestha A , Singh J. Inter-hospital and intra-hospital patient transfer: recent concepts. Indian J Anaesth. 2016;60(7):451–457. doi: 10.4103/0019-5049.186012. 27512159 PMC4966347

[47] Shinozaki RM , Schwingshackl A , Srivastava N , Grogan T , Kelly RB. Pediatric interfacility transport effects on mortality and length of stay. World J Pediatr. 2021;17(4):400–408. 34319538 10.1007/s12519-021-00445-w PMC8363522

[48] Aledhaim A , Hirshon JM , Fishe J , Anders J. Conditions Requiring Emergent Pediatric Interfacility Transport: Analysis of a Statewide EMS System. IL, USA: American Academy of Pediatrics Elk Grove Village; 2018.

[49] Dall’Ora C , Rubbo B , Saville C , et al. The association between multi-disciplinary staffing levels and mortality in acute hospitals: a systematic review. Hum Resour Health. 2023;21(1):30. doi: 10.1186/s12960-023-00817-5. 37081525 PMC10116759

[50] Siverskog J , Henriksson M. The health cost of reducing hospital bed capacity. Soc Sci Med. 2022;313:115399. doi: 10.1016/j.socscimed.2022.115399. 36206659

